# Supplementary material for: Plant stem cell maintenance involves direct transcriptional repression of differentiation program
Source: Mol Syst Biol. 2013 Apr 2;9:654. doi: 10.1038/msb.2013.8 (PMC3658276; doi:10.1038/msb.2013.8)
Supplement: Supplementary Information — Supplementary text describing computational methods together with Supplementary Figures [file msb20138-s1.pdf]

Supplementary Information
for

Plant stem cell maintenance involves direct transcriptional repression of differentiation program

R K Yadav, M Perales, J Gruel, C Ohno, M G Heisler, T Girke, H Jönsson, and G V Reddy

Contents

1 Supplementary Information **2**

2 Supplementary Figures (1-13) **10**

3 Supplementary Tables (1-8) **23**

4 Supplementary Movies (1-2) **31**

1 Supplementary Information

Template for geometry and expression domains

The shoot apical meristem is described as the top third of a sphere filled with 1366 overlapping spherical cells. Cells are regarded as neighbors (with transport in-between) if they overlap. Data files for cell positions and neighborhood information are available upon request. A binary expression template for the central zone (*CLV3*), organizing center (*WUS*) and peripheral zone (*KAN1*) was manually created based on experimental microscopy data (*cf.* Figure 5C-E in main text and [1, 2]). Three domains are defined as:

- the central zone (CZ): according to the expression of *CLV3*, is located at the tip of the meristem and comprises 55 cells.
- the organizing center (OC): according to the expression of *WUS*, is located below the CZ and comprises 42 cells.
- the (outer) peripheral zone (PZ): according to the expression of *KAN1*, is located in the outer layer of the meristem, starting 5 to 6 cells below the CZ and comprises 267 cells.

Model

To get an understanding of how the experimental data in the paper fits in the context of stem cell regulation interactions in the SAM, we implemented an ordinary differential equation model including a gene regulatory network within individual cells as well as signalling between cells and made simulations on a three-dimensional tissue template approximating the geometry and topology of the shoot apical meristem (Figure 5C-E in the main text). The model comprises the mRNA (expression) and proteins of *WUS*, *CLV3*, *KAN1* as representatives of OC, CZ, and PZ expressed genes, respectively.

Regulation of *WUSCHEL* expression In the model, *WUS* is repressed by a diffusive CLV3 signal [3, 4] (which is a simplification of the repressive signal generated by the CLV3 ligand binding to CLV1 and other receptors). A localized *WUS*-activation is used as suggested by the localized expression of the cytokinin (known to activate *WUS* expression) receptor AHK4 [5], as well as previous modeling efforts [6, 7]. Finally, ubiquitous expression of *KAN1* arrests the meristem [8], suggesting a repression of *WUS* by KAN1. This interaction is discussed in more details in section "*KAN1* effect on the core *WUS-CLV3* network".

Regulation of *CLAVATA3* expression *CLV3* is transcriptionally activated directly by the diffusing WUS [7] and by a hypothetical L1-originating signal used as a co-factor (as used and motivated in previous modeling efforts [9, 7]). The principal motivation for using a L1 signal as a co-activator is the striking *pCLV3::WUS* phenotype observed in [10], where both *CLV3* and *WUS* are expressed in the three superficial layers of the meristem.

Regulation of *KANADI1* expression The main text shows that *KAN1* counts as one of the peripheral zone genes directly repressed by the WUS transcription factor. In addition, the expression of *KAN1* remains located close to the L1 cell layer of the meristem, even when WUS diffusion is blocked, suggesting some activation of *KAN1* by a hypothetical L1 signal. The motivation for the L1 activation of *KAN1* is the observation that both in *wild type* and perturbed meristems, when *KAN1* is driven by its own promoter its expression domain remains confined to the superficial cell layers.

Anchors Localized *WUS* and epidermal *CLV3* and *KAN1* co-activation is implemented using two, in the model, constant 'anchors'. The first one (A1 - representing the activated AHK4 receptors), is situated in the organizing center and the second (A2 - representing the source of hypothetical epidermal signals) in the superficial cell layer (L1) (*cf.* Supplementary Figure 4). Both anchors are modeled as genes, *i.e.* a chimeric

gene is expressed resulting in the production of a signaling molecule that diffuses away from its production site, creating a concentration gradient. For simplicity, the values of A1 and A2 remain constant throughout the simulations and are set to one (note that possible equations to dynamically generate these patterns have previously been described [9, 6]).

We used a Hill description for transcriptional regulation and a linear dependence on RNA levels for protein production. RNA and proteins are degraded proportional to their concentration. Movement of proteins is implemented by a concentration-dependent (passive) diffusion-like transport.

The general equation for gene expression is:

$$\frac{d[X]}{dt} = V \times \prod^{A} \frac{[A]^n}{k_A^n + [A]^n} \times \prod^{I} \frac{k_I^n}{k_I^n + [I]^n} - g[X]$$

with $\frac{d[X]}{dt}$ the time derivative of the mRNA concentration, V the maximal rate of the considered gene expression (mRNA production), $[A]$ the concentrations of the gene activators and $[I]$ the concentrations of the inhibitors. The Hill constants k_A, k_I (setting up the concentration of activators/inhibitors necessary for the gene to switch between regimes of maximal and minimal expression) and the Hill coefficient, n (always set to 2, controls the steepness of the gene expression switch), regulate the effect of their respective activators and inhibitors in individual cells. The mRNAs undergo a degradation proportional to g.

The general equation handling protein behavior is:

$$\frac{d[x]}{dt} = P[X] - g[x] + D\Delta[x]$$

with X the mRNA counterpart of the x signaling molecule (protein or peptide), P the production rate - proportional the mRNA concentration, g the degradation rate and D the diffusion rate. Δ is the Laplace operator; transport of signaling molecules in the model is assumed to be passive, and geometrical factors are set to one, *i.e.* the contribution to the derivative for a molecule in cell i is given by $\sum_n([x]_n - [x]_i)$, where the sum is over neighboring cells.

In the following, mRNAs for *CLV3*, *WUS* and *KAN1* will be referred as C, W and K while the corresponding signaling molecules will be referred as c, w and k. a1 (a1c/a1k) and a2 will refer to the chimeric signaling molecules produced by anchors A1 and A2, respectively. The model is described by

$$\begin{aligned}
\frac{d[C]}{dt} &= V_C \times \frac{[a1c]^n}{k_{a1c/C}^n + [a1c]^n} \times \frac{[w]^n}{k_{w/C}^n + [w]^n} - g_C[C] \\
\frac{d[c]}{dt} &= P_c[C] - g_c[c] + D_c\Delta[c] \\
\frac{d[W]}{dt} &= V_W \times \frac{[a2]^n}{k_{a2/W}^n + [a2]^n} \times \frac{k_{c/W}^n}{k_{c/W}^n + [c]^n} \times \frac{k_{k/W}^n}{k_{k/W}^n + [k]^n} - g_W[W] \\
\frac{d[w]}{dt} &= P_w W - g_w[w] + D_w\Delta[w] \\
\frac{d[K]}{dt} &= V_K \times \frac{[a1k]^n}{k_{a1k/K}^n + [a1k]^n} \times \frac{k_{w/K}^n}{k_{w/K}^n + [w]^n} - g_K[K] \\
\frac{d[k]}{dt} &= P_k[K] - g_k[k] + D_k\Delta[k] \\
\frac{d[a1c]}{dt} &= P_{a1c}[A1] - g_{a1c}[a1c] + D_{a1c}\Delta[a1c] \\
\frac{d[a1k]}{dt} &= P_{a1k}[A1] - g_{a1k}[a1k] + D_{a1k}\Delta[a1k] \\
\frac{d[a2]}{dt} &= P_{a2}[A2] - g_{a2}[a2] + D_{a2}\Delta[a2]
\end{aligned}$$

Model parameters

Since model parameters have unknown values, we applied an optimization strategy to extract parameter values where the model outputs were able to fit the experimentally based expression domains of *WUS*, *CLV3* and *KAN1* on the three-dimensional tissue template [7, 11]. An analytical study of equilibrium equations was conducted, which showed that the variation of a subset of parameters can produce all the possible behaviors. These parameters were updated in the optimization procedure (Supplementary Table 8).

A stochastic gradient descent and exploration algorithm was implemented to minimize a cost function defined by the square difference between the template and the equilibrium from the simulation of *WUS*, *CLV3* and *KAN1* mRNA levels summed over all cells [12, 7]. The algorithm is initiated with random values for parameters to be optimized and the cost function is computed after the model is simulated until equilibrium. One of the parameters is then randomly selected and its value is randomly increased or decreased by a fixed factor. The cost function is computed from a simulation using the new set of parameters. If the cost function value increases, the probability of modifying the same parameter in the same direction is decreased. If the value of the cost function decreases, the probability of taking the same step is increased. The gradient of the cost function and the optimal set of parameters is approached by repeating the procedure and updating the value of the parameters along with the probability to select and change a parameter in a given direction. Typically the algorithm was run for 10.000 steps and a parameter was increased or decreased by one percent of its previous value at each step, the initial values of parameters were randomly generated between 0 and 0.1 (uniformly). Once the threshold, t_c, of the cost function is attained ($t_c = 60$ was used), sets of parameters are considered as solutions. While the cost is below t_c, a parameter exploration is continued without updating the probabilities for selecting directions, and one set of parameters out of a thousand is stored for further analysis.

This optimization strategy resulted in 229 sets of parameters properly describing the observed gene expressions. An example of the model output for one selected set of parameter values is shown in Figure 5C-F in the main text and the values of the parameters are given in Supplementary Table 8.

Perturbations

We investigated the model behavior for several perturbations (17 in total). Out of all the tested perturbations, the model appear to qualitatively follow what was observed in experiments where these are available, and in addition the different parameter values gave consistent results (Table 1 in Main text, Supplementary Figure 7 and 12), showing a robust behavior for the model network. Below, the modifications applied to the model at each perturbation are described. In all cases, the perturbations are applied to the model for all the sets of parameters obtained from the optimization, after it reached its stable (equilibrium) state.

Loss-of-function The production of one of the proteins is decreased by a factor 10 (3 perturbations: *CLV3*, *WUS*, *KAN1*).

- Using *pCLV3::GUS* transfected *wus-1* plants, [10] shows the expression of *CLV3* drops to undetectable levels in mature embryos, this behavior is mirrored by all the model sets of parameters (Supplementary Figure 7a) and further exemplified in Supplementary Figure 3b where *CLV3* expression is undetectable. In addition the model predicts an expansion of the *KAN1* expression domain.

- A Dex-inducible *clv3* phenotype is described in [2]. Imaging shows expansion of both the *CLV3* and *WUS* expression domains. Application of the mutation to the computer model shows that the amount of both *WUS* and *CLV3* mRNA increases for the all the optimized sets of parameters, a reduction of the *KAN1* expression domain is also predicted (Supplementary Figure 7b). The expansion of the CLV3 domain is exemplified in Supplementary Figure 3c.

- *KAN1* loss-of-function is described in [8] as having no noticeable effect on the SAM, and applying this perturbation to the model resulted in no effect.

Over-expression The production of one of the proteins (signaling molecules) is increased by a factor 10 (3 perturbations: *CLV3*, *WUS*, *KAN1*). Over-expression of *KAN1* did not have any effect on the model behavior.

- for *WUS*, the model predicts a decrease of *WUS* promoter activity, along with an increase of *CLV3* expression domain and an decrease of *KAN1* expression domain.

- such perturbations have been investigated in [13] and [14]. While a slight overexpression of *CLV3* does not appear to yield any difference in the system, consistently with the model, an important overexpression leads to a decrease of *WUS* promoter activity. Additionally, the model predicts a decrease of the native *CLV3* expression, and an increase of *KAN1* expression.

The behavior of the mutants in the simulations is the opposite of what is observed for loss-of-function mutants, *i.e.* when the expression of a gene increases in a loss-of-function settings it decreases in its over-expression counterpart and *vice versa* (see Supplementary Figures 7c,d and 3m,n).

Ubiquitous expression In addition to its normal expression, one gene is constantly expressed in every cell. The rate of this production is twice the rate of the mRNA degradation (3 perturbations: *CLV3*, *WUS*, *KAN*). This simulations are designed to emulate the expression of a gene under the control of the *CaMV* (cauliflower mozaic virus) *35S* promoter.

- Using Dex inducible *p35S::WUS* plants in [15], it was shown that the ubiquitous expression of *WUS* resulted in a dramatic expansion of the *CLV3* expression domain. A corresponding effect of the mutation is also observed with all the sets of parameters, along with an equally dramatic reduction of *KAN1* expression domain (Supplementary Figure 7e and example in Supplementary Figure 3d).

- SAM expressing *p35S::CLV3* constructions were observed in [13]. As observed in the corresponding simulations (Supplementary Figure 7f and example in Supplementary Figure 3e), no *WUS* mRNA was detectable. Another experiment using this construct is described in the main text. It shows a gradual expansion of the *KAN1* expression domain towards the Central Zone, as observed in Supplementary Movie 1. Although the expansion of the domain stops at the boundary with the Central Zone, the model predicts a complete coverage of the meristem. A few hypothesis can explain this scenario: the construction does not completely repress *WUS* expression (an example is presented in Figure 10, where clv3 peptide is ubiquitously produced, but to a lower level), the complete transformation happens after 96h and is thus not captured by the experimental settings, or some additional WUS-independent mechanism prevents a complete transformation.

- Observing large perturbations of seedlings expressing *p35S::KAN1*, the authors of [8] suggest that they would also lack a SAM. This is coherent with the results obtained from the simulations in which, for all parameter sets, expression of both *WUS* and *CLV3* is largely inhibited (Supplementary Figure 7g and example in Supplementary Figure 3f). This perturbation is further discussed in section "Effect of *KAN1* on the core *WUS-CLV3* network", additional data is also provided in Supplementary Figure 5 showing a decrease of *WUS* expression.

Mis-expression These perturbations mimic plants transfected with a construction including the promoter of one gene and the cDNA of another. In addition to its normal expression, one gene is expressed in the same location and amount as another (6 perturbations: *pWUS::CLV3*, *pWUS::KAN*[this work] and *pCLV3::WUS*[10], *pCLV3::KAN1*, *pKAN1::WUS* and *pKAN1::CLV3* (Supplementary Figures 12a-f and 3g-l)

- for *pWUS::CLV3*, the model predicts an decrease of *WUS* expression and an increase of *KAN1* expression.

- the *pWUS::KAN1* case is described in the main text and results in meristem arrest. Consistent with this observation, the corresponding simulations result in large decreases of *WUS* and *CLV3* expressions.

- *pCLV3::WUS* is described in [10]. In addition to the formation of a large apical dome, *WUS* and *CLV3* are strongly expressed in the 3 outer cell layers of the SAM. With all the sets of parameters, an increase of *WUS* and *CLV3* expression is observed, along with a reduction of *KAN1* expression. As exemplified in Supplementary Figure 3i, *WUS* and *CLV3* are also expressed in the 3 outer cell layers.
- for *pCLV3::KAN1*, the model predicts a decrease of both *WUS* and *CLV3* expression.
- for *pKAN1::WUS*, the model predicts an increase of *CLV3* expression along with a decrease of *KAN1* expression
- for *pKAN1::CLV3*, the model predicts a decrease of *WUS* expression along with an increase on *KAN1* expression

Transport malfunction mutants The diffusion rate of WUS is reduced to 0. This simulation mirrors the experiments described in [7], in which WUS is fused with 2 GFPs or a NLS in order to restrict its movement between cells. The two constructions are described as being unable to rescue *wus-1* mutants. Consistent with this observation, the corresponding simulations show an important reduction of *CLV3* expression, along with an increase of *KAN1* expression (Supplementary Figures 12g and 3o). The same perturbation was also applied to *CLV3*. The simulations predicts an increase of *WUS* expression along with a decrease of *KAN1* expression. The variation of *CLV3* expression varies depending on the sets of parameters (Supplementary Figure 12h). As the model does not include CLV3 receptors, CLV3 acts not only on the cells it reaches while diffusing, but also on the very same cells it is produced in (without leaving the cell). This behavior, along with these simulations results, might not be a valid description of biological reality.

Growth and cell division

In order to assess the robustness of the three gene expression domains to changes in cellular topology, the model was run with settings including cell growth and division [16, 17].

Supplementary Movie 2 uses the parameters described in Supplementary Table 3. The cells of the template are enclosed in a lid shaped as the third of a sphere. Cells are allowed to grow by increasing the cell radii and divide in two daughter cells when a threshold size is attained. A set of spring forces between cell centers moves cells appart when overlapping and back inside the lid when pushed out of it. Cells falling below the lower boundary of the lid are removed from the simulation. The two anchors are adapted to the dynamical conditions while the equations describing RNAs and proteins remain unchanged. Finally a set of variables and are added to account for cell divisions, spring forces and diffusion as the cell neighborhood varies over the simulation.

The movie shows that the expression domains of the three studied genes perfectly withstand the perturbations induced by the changing cell layout and remain largely constant while cells enter and exit the expression regions. Due to the heavy computations required for this type of simulation, it was performed only with this set of parameters.

Minimality

Our results show that the model is sufficient to describe both the correct gene expression patterning of the wild type meristem, and also known perturbations. Additionally, some experiments were conducted in order to see whether this model hosts any unnecessary interactions for achieving these results.

To check if the desired behavior could be achieved with reduced models, the optimization procedure described above was applied to a set of altered models.

In a first round of optimizations a model handling only the CZ and OC was used, a model able to describe both wild type and perturbations of *WUS* and *CLV3* expression ([7]). Drawn from this model, 4 altered models were designed, each one having one interaction less than the parent model. Out of these reduced models, and following an optimization procedure that ensured finding solutions for the parent model, only the model without the repression by CLV3 on *WUS* achieved to get close to describing the correct gene

expression patterns of the wild type meristem. Further testing showed that applying a *clv3* loss-of-function mutation to solutions of this model does not have any effect. Those 4 models were thus discarded as potential descriptions of the gene expression in the SAM.

Second, we tested the remaining interactions with altered versions of the model including *KAN1* expression. Three additional models were designed, each one without one of the interactions involving *KAN1*. Loosing the L1 activation or the WUS repression of KAN1 leads to mis-positioned KAN1 expression domain, discarding the corresponding models. Finally, the *wild type* is correctly represented if the repression of KAN1 on WUS is not present, also for the vast majority of the perturbations results are similar to the full model. However, the ubiquitous expression of KAN1 or its mis-expression in the Organizing Center fail to induce a response of *WUS* or *CLV3*, leading to the inclusion of this interaction in the final model.

These experiments show that the model presented here represents a sufficient and minimal set of interactions required to describe the spatial orgaization of OZ, CZ and PZ in the SAM.

Sensitivity analysis

In order to analyse the sensitivity of the model to variations of its parameters, the value of the 37 parameters of the model have been increased by 1 percent. For each set of parameters and each parameter, the sensitivity has been computed following: $\frac{\Delta m}{\Delta p} \times \frac{p}{m}$, with p and Δp, the original parameter value and its variation and with m and Δm, the measure and its variation. In Supplementary Figure 13, the measure refers to the total expression of *WUS*, *CLV3* or *KAN1* and how this depends on all model parameters. In genereal, a robust behavior of the model to those perturbations was observed.

It appears that *WUS* and *CLV3* expression are on overall mostly sensitive to the same parameters, the parameters controlling their expression and the parameters controlling their anchors. Parameters controlling *KAN1* expression have a negligible impact on those two genes.

KAN1 expression is sensitive to variations of most of the parameters of the model, but especially to variations of those controlling its own expression and its anchoring.

KAN1 effect on the core *WUS-CLV3* network

As shown in [8] an ubiquitous expression of *KAN1* leads to a meristem arrest. Within the modeling framework presented here, we consider the meristem arrested when the expression of *CLV3* or *WUS* is lost or dramatically perturbed. Considering the expression domain of *KAN1* and the data presented in Supplementary Figures 11 and 5, we suppose that *KAN1*, likely in an indirect manner, leads to a loss of expression of *CLV3* or *WUS* where it is expressed. Three options can thus be considered to explain the arrest of a meristem harbouring a *35S::KAN1* construct.

- *KAN1* represses *WUS* (hypothesis h1, presented in the main text)
- *KAN1* represses *CLV3* (hypothesis h2)
- *KAN1* represses both *WUS* and *CLV3* (hypothesis h3)

Intuitively, in the h1 case, the loss of *WUS* induces a loss of *CLV3*. In the h2 case the loss of *CLV3* induces an overexpression of *WUS*, due to the loss of clv3 repression of *WUS*. Finally in the h3 case, one expects to observe a loss of expression of the 2 genes.

If generally true, the latter scenario can produce more complex dynamics. If the repression of *WUS* by *KAN1* requires a more important *KAN1* concentration to occur than the repression of *CLV3* by *KAN1*. One can observe a transitory overexpression of *WUS* due to the early loss of *CLV3*. As the concentration of *KAN1* builds up in cells, *WUS* is eventually repressed.

Example dynamics of *WUS* and *CLV3* concentration upon ubiquitous expression of *KAN1* are shown in Supplementary Figure 6.

Finally, although hypothesis h1 and h3 can explain the loss of *WUS* expression upon ubiquitous expression of *KAN1* shown in Supplementary Figure 5 (as well as the arrested meristem of Supplementary Figure 11), the most parsimonious hypothesis, presented in the main text, is a repression of kan1 on *WUS*. It is to be

noted that this repression might not be a direct effect, we consider more likely that the expression of *KAN1* in the rib meristem induces a cellular differentiation program resulting in the loss of *WUS* expression.

Implementation

All simulations were done using the Organism simulation package (http://dev.thep.lu.se/organism) using a 5th order Runge-Kutta solver with adaptative step size. Optimization and analysis scripts are in-house software developed in Python. All used software is available upon request, together with parameters and initial condition files.

References

[1] Yadav, R. K., Girke, T., Pasala, S., Xie, M., and Reddy, G. V. Gene expression map of the Arabidopsis shoot apical meristem stem cell niche. *Proc. Natl. Acad. Sci. U.S.A.* **106**, 4941–4946, Mar (2009).

[2] Reddy, G. V. and Meyerowitz, E. M. Stem-cell homeostasis and growth dynamics can be uncoupled in the Arabidopsis shoot apex. *Science* **310**, 663–7 (2005).

[3] Sharma, V. K., Carles, C., and Fletcher, J. C. Maintenance of stem cell populations in plants. *Proc Natl Acad Sci USA* **100 Suppl 1**, 11823–9 (2003).

[4] Ogawa, M., Shinohara, H., Sakagami, Y., and Matsubayashi, Y. Arabidopsis CLV3 peptide directly binds CLV1 ectodomain. *Science* **319**, 294 (2008).

[5] Gordon, S. P., Chickarmane, V. S., Ohno, C., and Meyerowitz, E. M. Multiple feedback loops through cytokinin signaling control stem cell number within the Arabidopsis shoot meristem. *Proc Natl Acad Sci USA* **106**, 16529–34 (2009).

[6] Jönsson, H., Heisler, M., Reddy, G. V., Agrawal, V., Gor, V., Shapiro, B. E., Mjolsness, E., and Meyerowitz, E. M. Modeling the organization of the WUSCHEL expression domain in the shoot apical meristem. *Bioinformatics* **21 Suppl 1**, i232–40 (2005).

[7] Yadav, R. K., Perales, M., Gruel, J., Girke, T., Jönsson, H., and Reddy, G. V. WUSCHEL protein movement mediates stem cell homeostasis in the Arabidopsis shoot apex. *Genes Dev.* **25**, 2025–2030 (2011).

[8] Kerstetter, R. A., Bollman, K., Taylor, R. A., Bomblies, K., and Poethig, R. S. KANADI regulates organ polarity in Arabidopsis. *Nature* **411**, 706–9 (2001).

[9] Jönsson, H., Shapiro, B., Meyerowitz, E., and Mjolsness, E. Signaling in multicellular models of plant development. In On Growth, Form and Computers, Kumar, S. and Bentley, P., editors, 156–161. Academic Press, London, Jan (2003).

[10] Brand, U., Grünewald, M., Hobe, M., and Simon, R. Regulation of CLV3 expression by two homeobox genes in Arabidopsis. *Plant physiology* **129**, 565–75 (2002).

[11] Sahlin, P., Melke, P., and Jönsson, H. Models of sequestration and receptor cross-talk for explaining multiple mutants in plant stem cell regulation. *BMC systems biology* **5**, 2 (2011).

[12] Gruel, J., Leborgne, M., Lemeur, N., and Theret, N. In silico investigation of ADAM12 effect on TGF-beta receptors trafficking. *BMC Res Notes* **2**, 193 (2009).

[13] Brand, U., Fletcher, J. C., Hobe, M., Meyerowitz, E. M., and Simon, R. Dependence of stem cell fate in Arabidopsis on a feedback loop regulated by CLV3 activity. *Science* **289**, 617–9 (2000).

[14] Muller, R., Borghi, L., Kwiatkowska, D., Laufs, P., and Simon, R. Dynamic and compensatory responses of Arabidopsis shoot and floral meristems to CLV3 signaling. *Plant Cell* **18**(5), 1188–1198, May (2006).

[15] Yadav, R. K., Tavakkoli, M., and Reddy, G. V. WUSCHEL mediates stem cell homeostasis by regulating stem cell number and patterns of cell division and differentiation of stem cell progenitors. *Development* **137**, 3581–9 (2010).

[16] Jönsson, H., Shapiro, B., Meyerowitz, E., and Mjolsness, E. Modeling plant development with gene regulation networks including signaling and cell division. In Bioinformatics of geneome regulation and structure, Kolchanov, N. and Hofestaedt, R., editors, 311–318. Kluwer Academic Publishers, Boston (2004).

[17] Jönsson, H., Heisler, M. G., Shapiro, B. E., Meyerowitz, E. M., and Mjolsness, E. An auxin-driven polarized transport model for phyllotaxis. *Proc Natl Acad Sci USA* **103**, 1633–8 (2006).

2 Supplementary Figures (1-13)

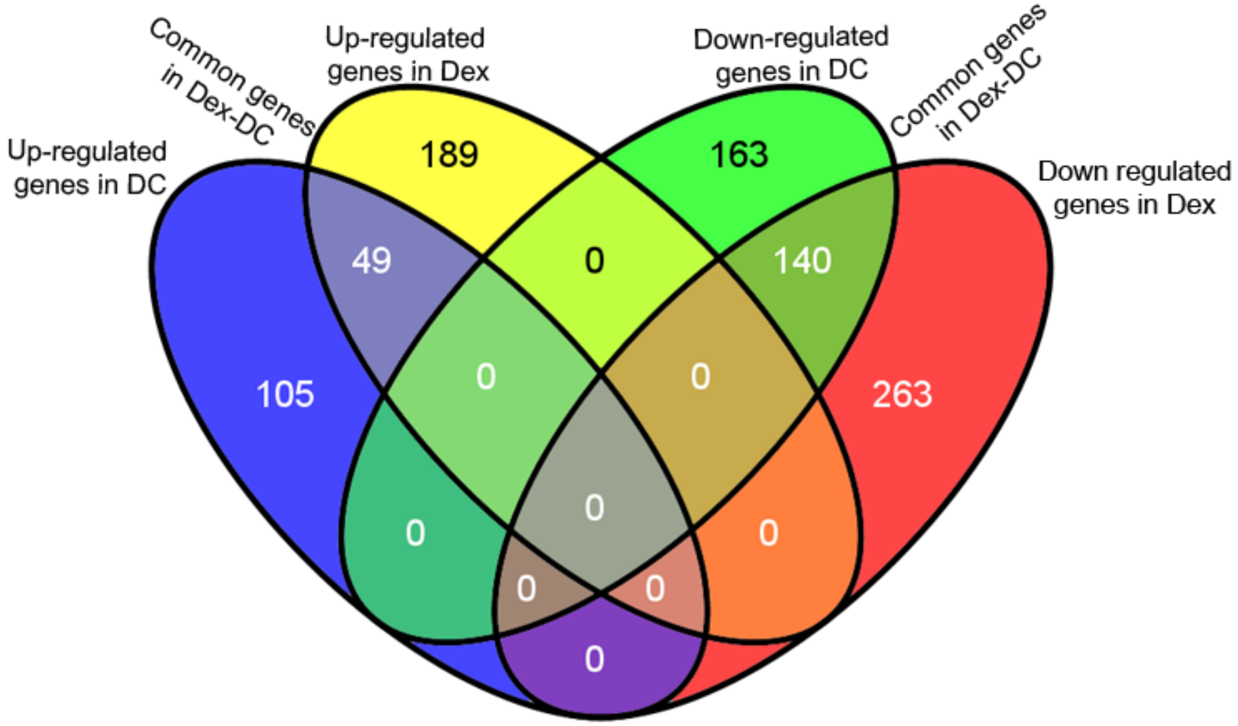

Supplementary Figure 1: **Four-way Venn diagram showing the distribution of *WUS* up and down regulated genes in Dex treatment alone and in Dex plus Cyc (DC) treatments.** Numbers within each sector represent number of *WUS*-responsive genes.

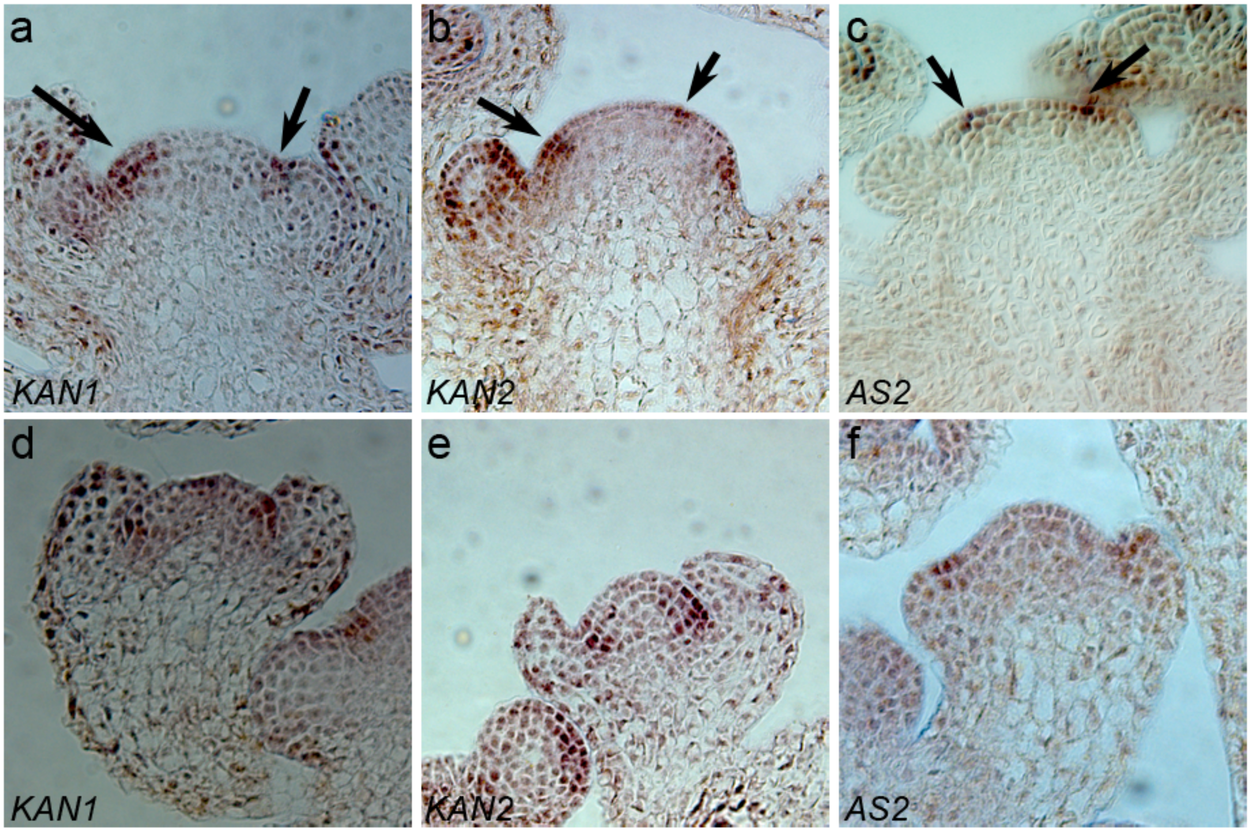

Supplementary Figure 2: **Expression patterns of differentiation promoting transcription factors.** RNA localization patterns of *KAN1* (a), *KAN2* (b) and *AS2* (c) in SAMs and *KAN1* (d), *KAN2* (e) and *AS2* (f) in developing flowers. Arrows indicate RNA accumulation in differentiating peripheral zone cells.

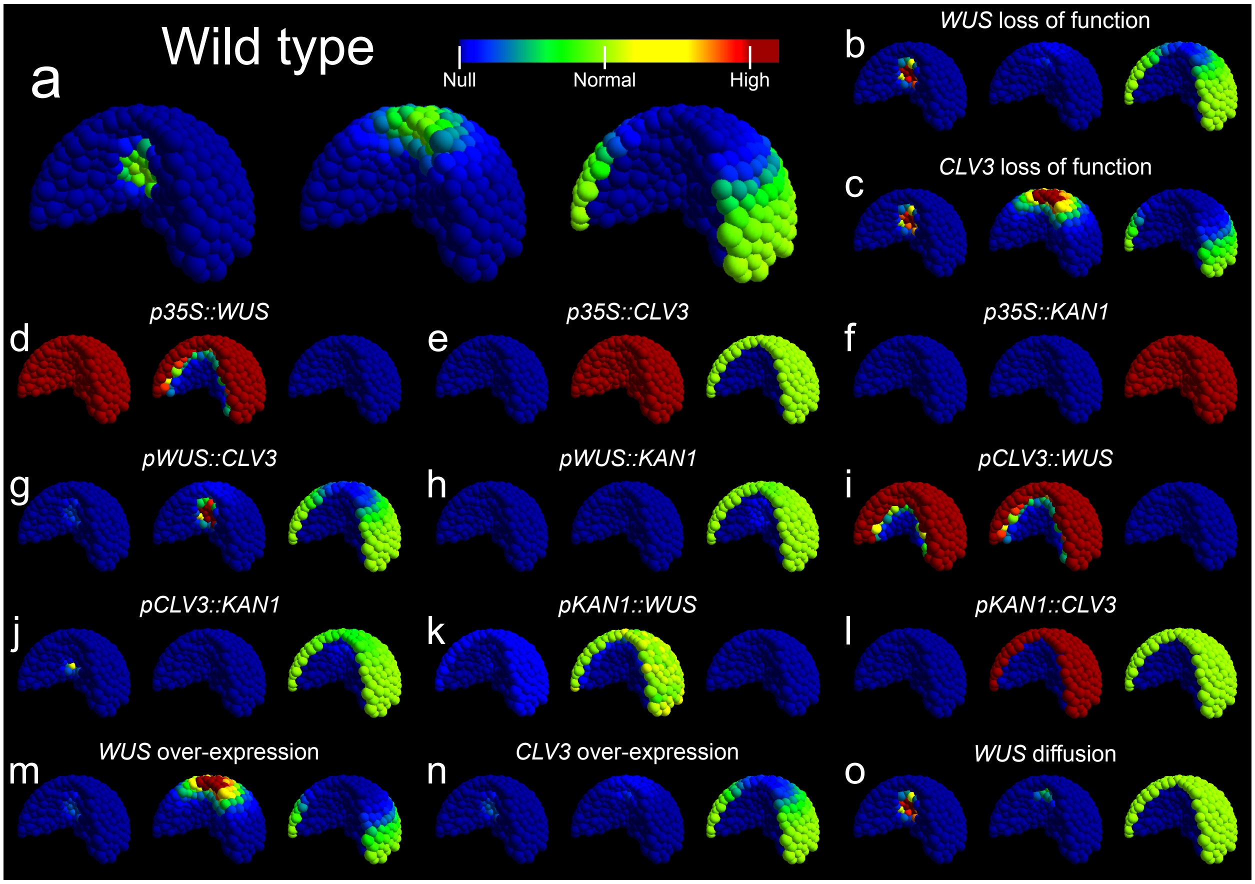

Supplementary Figure 3: **Mutant examples.** Perturbation behavior for the model using the parameter values from Supplementary Table 8 and perturbations as described in the Supplementary Information. Each sub-figure shows OZ (*WUS*, left), CZ (*CLV3*, middle), and PZ (*KAN1*, right) expressions. The color scale label 'normal' indicates the template defined expression level. a) Wild type. b)-c) loss-of-function mutants. d)-f) ubiquitous expression mutants. g)-l) mis-expression mutants. m)-n) over expression mutants (increased promoter strength). o) WUS transport malfunction mutant.

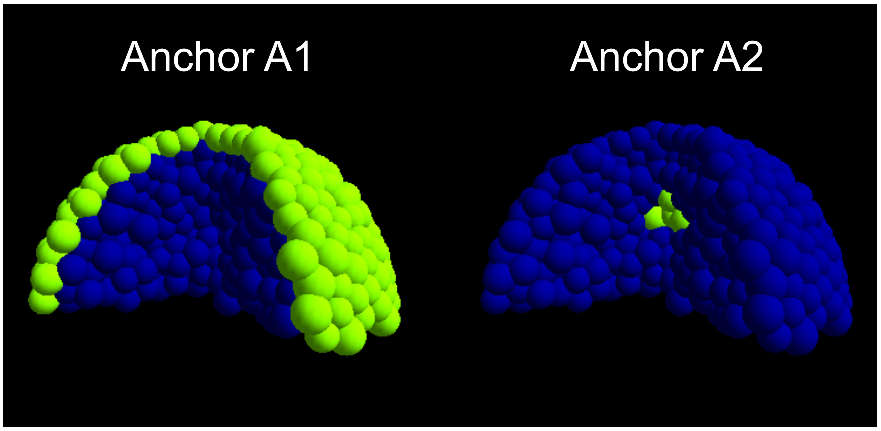

Supplementary Figure 4: **Position of the anchors.** Anchors for co-regulating *CLV3* and *KAN1* (left) and *WUS* (right) expression. These anchors are set to one in the green cells and kept constant throughout the simulations.

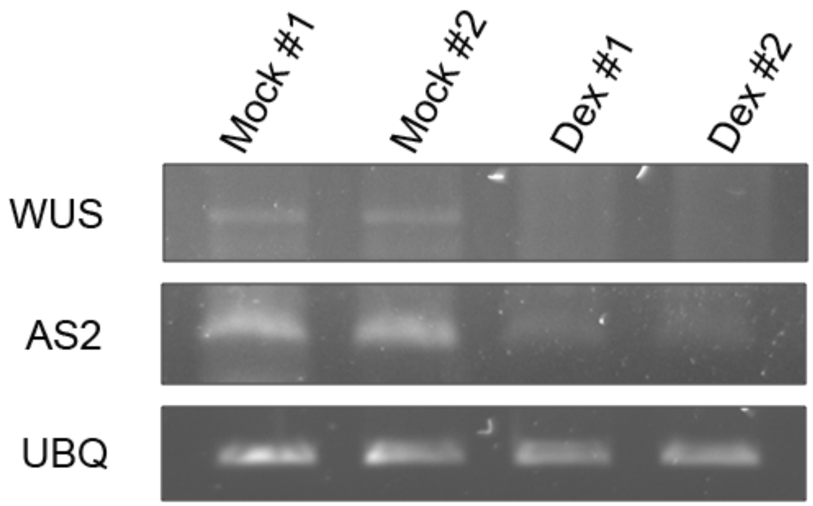

Supplementary Figure 5: **Constitutive expression of *KAN1* represses *WUS* expression.** RTPCR analysis of *WUS* and *AS2* (a known repressed target of *KAN1*) was performed on RNA extracted from finely dissected vegetative meristem of *35:KAN1-GR* seedlings grown on 10 uM Dex and Mock plates for five days. Two biological replicas were analyzed. Ubiquitin was used as internal reference. Note downregulation of both *WUS* and *AS2* expression.

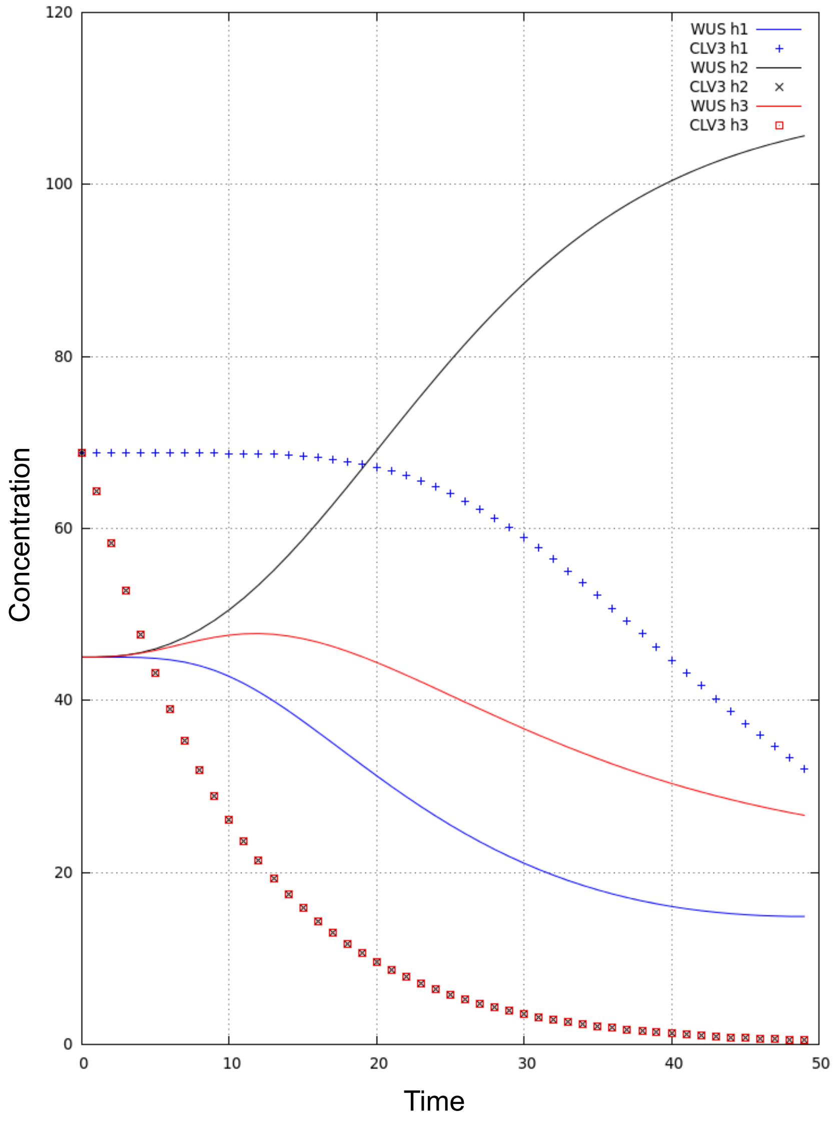

Supplementary Figure 6: **Effect of *KAN1* ubiquitous expression.** x axis represents time after ubiquitous expression of *KAN1* and y axis represents concentration of *WUS* and *CLV3* summed over all meristem cells, both are arbitrary units. Three hypothesis (h1, h2 and h3) are presented and described in section "*KAN1* effect on the core *WUS*-*CLV3* network". In h1 and h3: $k_{k/W} = 5$, in h2 and h3: $k_{k/C} = 0.01$. In h3, note the transitory overexpression of *WUS* due to $k_{k/W} >> k_{k/C}$.

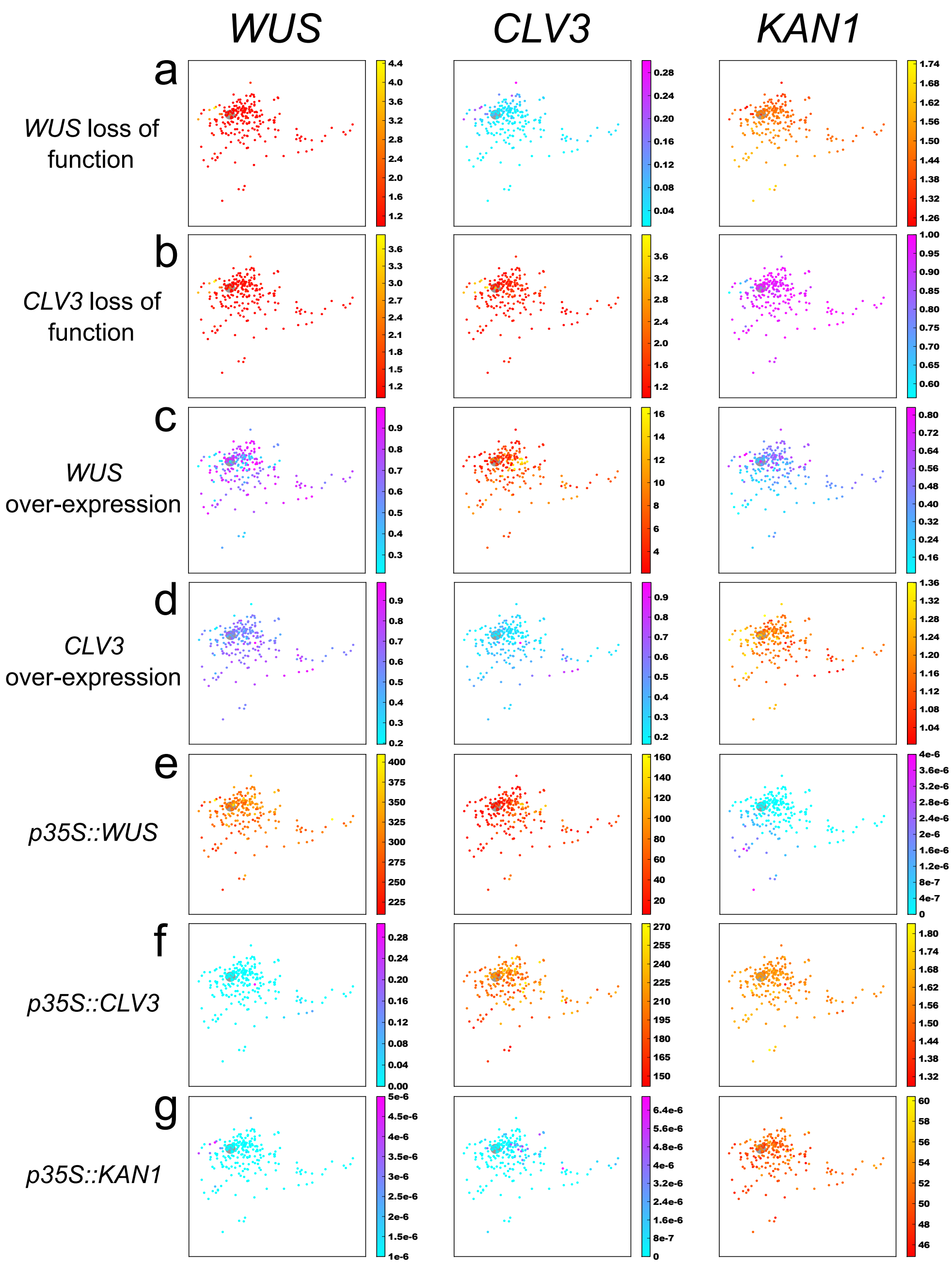

Supplementary Figure 7: **Loss-of-function and over-expression mutants.** Parameter sets are displayed using principal component analysis (first and second principal directions, *cf.* Figure 5b in main text). The color scale represents the variation of the expression of the considered gene between the wild type and the considered mutant. The three columns show the change in OZ/*WUS* (left), CZ/*CLV3* (middle), and PZ/*KAN1* (right) expression. The gray disc is centered on the parameter set exemplified in Supplementary Figure 3 (Supplementary Table 8). a) loss-of-function mutants for *WUS* and b) *CLV3*. c) overexpression (increased promoter activity) mutants for *WUS* and d) *CLV3*. E) Ubiquitous expression mutants of *WUS*, f) *CLV3*, and g) *KAN1*. See Supplementary Information for description of mutant implementations.

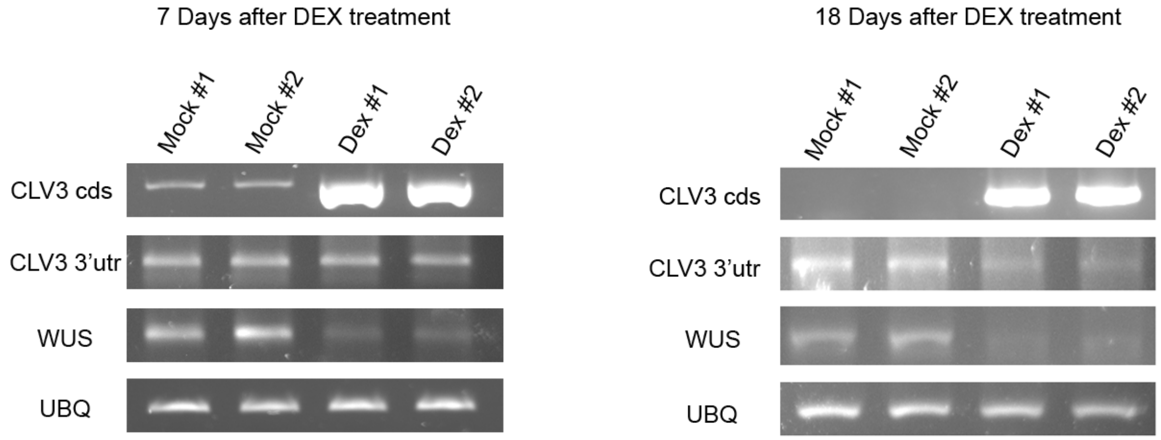

Supplementary Figure 8: **Effect of constitutive CLV3 overexpression on in vivo *CLV3* and *WUS* transcript levels.** RTPCR analysis was performed on RNA extracted on finely dissected meristems of 7 days and 18 days old *35S::GR-LhG4*;*6XoP::CLV3* seedlings grown on 10 uM Dex and Mock plates. Two biological replicas were analyzed. Ubiquitin was used as internal reference.

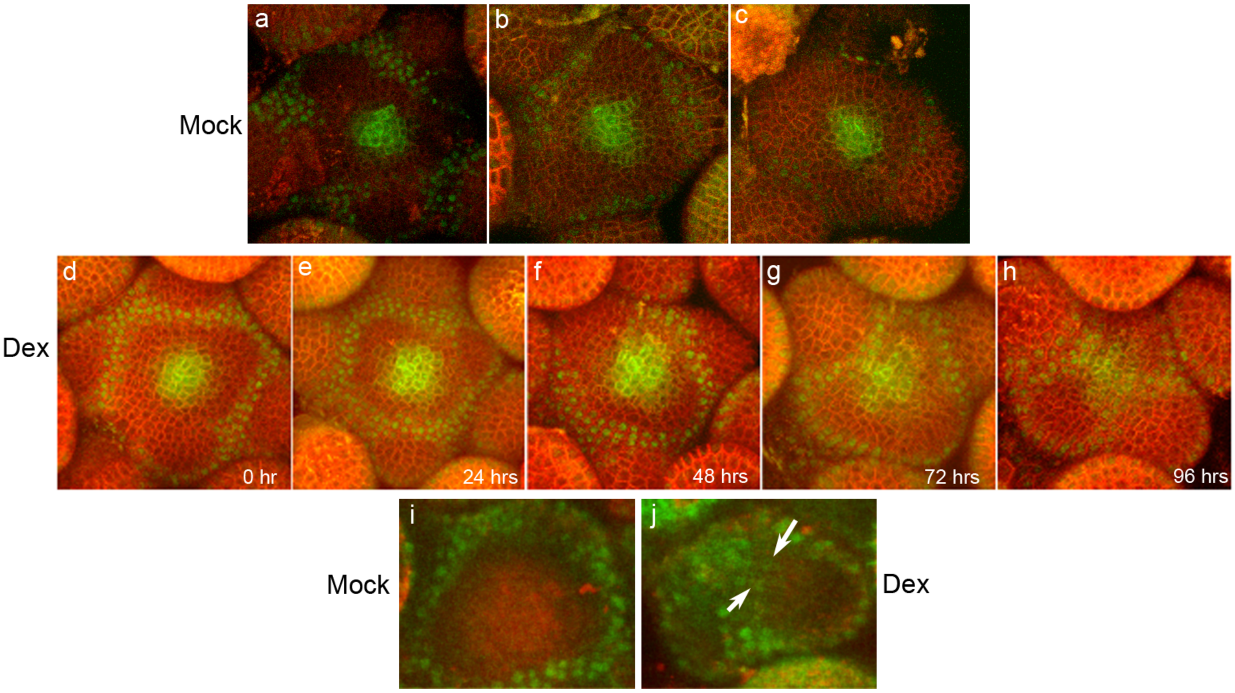

Supplementary Figure 9: **Premature differentiation of stem cell progenitors upon transient increase in *CLV3* levels.** (a-h) 3D-reconstructed top views of SAMs labeled with stem cell/CZ - *pCLV3::mGFP5-ER* [endoplasmic reticulum localized GFP] and the *pKANADI1::KANADI1:GFP* [nuclear localized GFP] reporter expressed in differentiating cells located at the outer edges of the PZ. (a-c) A time lapse series showing *pCLV3* and *KAN1* expression in mock-treated *35S::GR:LhG4; 6XOP::CLV3* plants and (d-h) dex- treated *35S::GR:LhG4*; *6XOP::CLV3* plants. Total time elapsed upon mock or dex treatment is indicated on each panel. (i) and (j) are mock- and dex-treated (for 5 days) *35S::GR:LhG4*; *6XOP::CLV3* plants respectively that carry only *pKAN1::KAN1:GFP* transgene (green). Arrows in (j) shows few *KAN1* expressing cells.

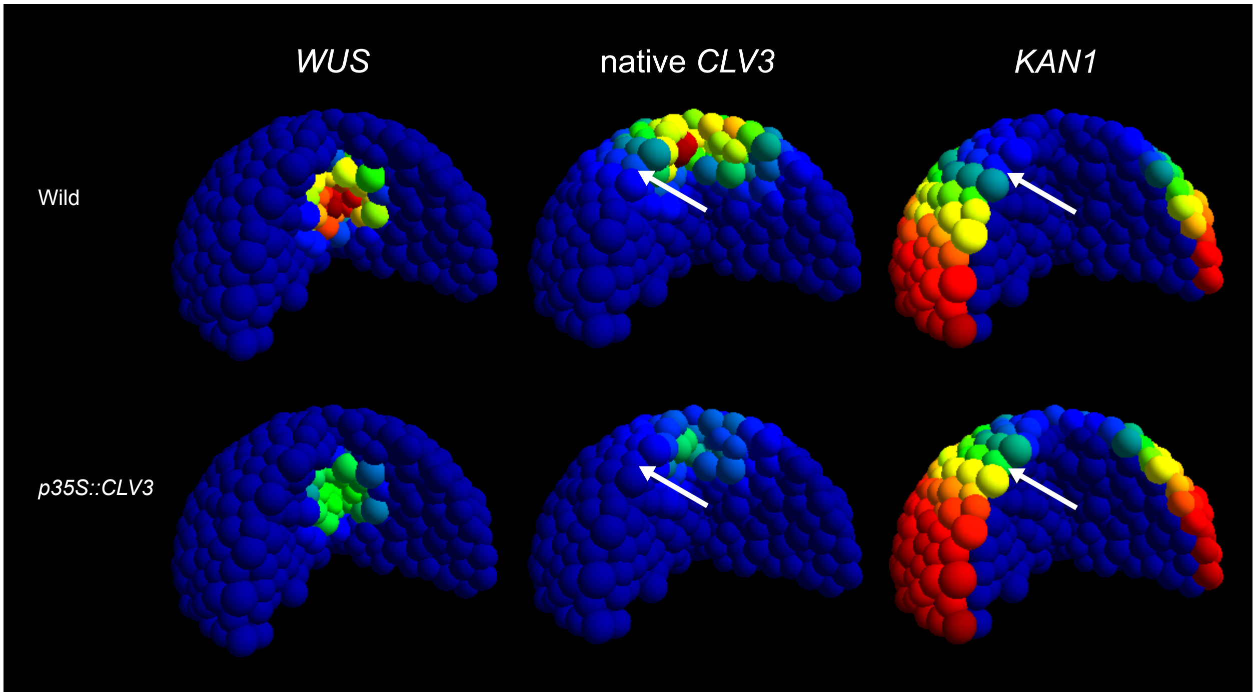

Supplementary Figure 10: ***p35S::CLV3.*** Comparison between wild type gene expression patterns and a *p35S::CLV3* construct (low efficiency). The colour scheme is relative to the highest and lowest concentrations in the simulation. The simulation is implemented so that the native *CLV3* is visible (central row). Left and right rows present *WUS* and *KAN1*, respectively. Top line shows wild type and bottom line shows the *p35S::CLV3* perturbed expressions. As presented in the counterpart experimental Supplementary Figure 9, the expression domain of *KAN1* extends towards the central zone, without covering it.

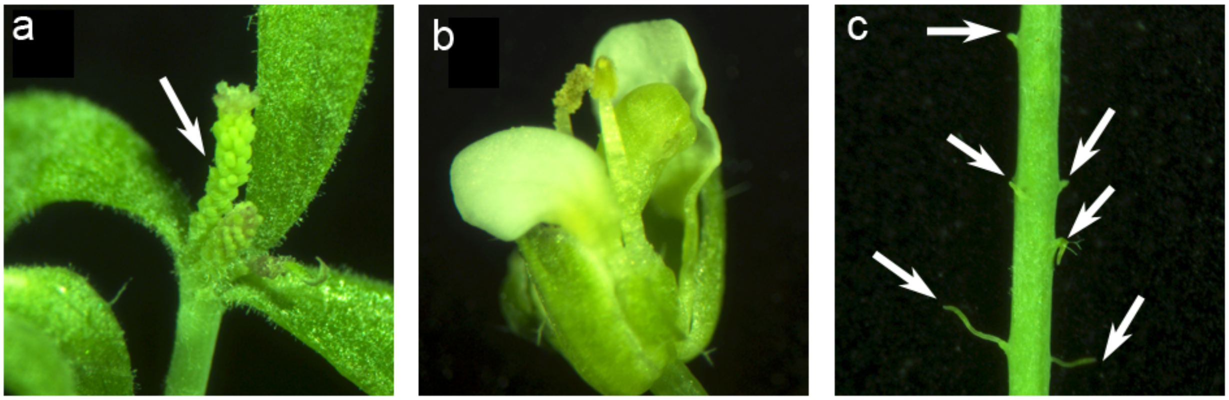

Supplementary Figure 11: **Mis-expression of *KANADI1* in the niche/organizing center (*WUS* expression domain) leads to shoot and floral meristem defects.** (a) A 25-day old plant showing SAM terminated with a cob-like inflorescence consisting of arrested floral buds. (b) a representative flower showing reduced floral organs. (c) Arrows indicate filamentous structures arranged on inflorescence stem. Pattern of arrangement suggests that they could be arrested floral buds.

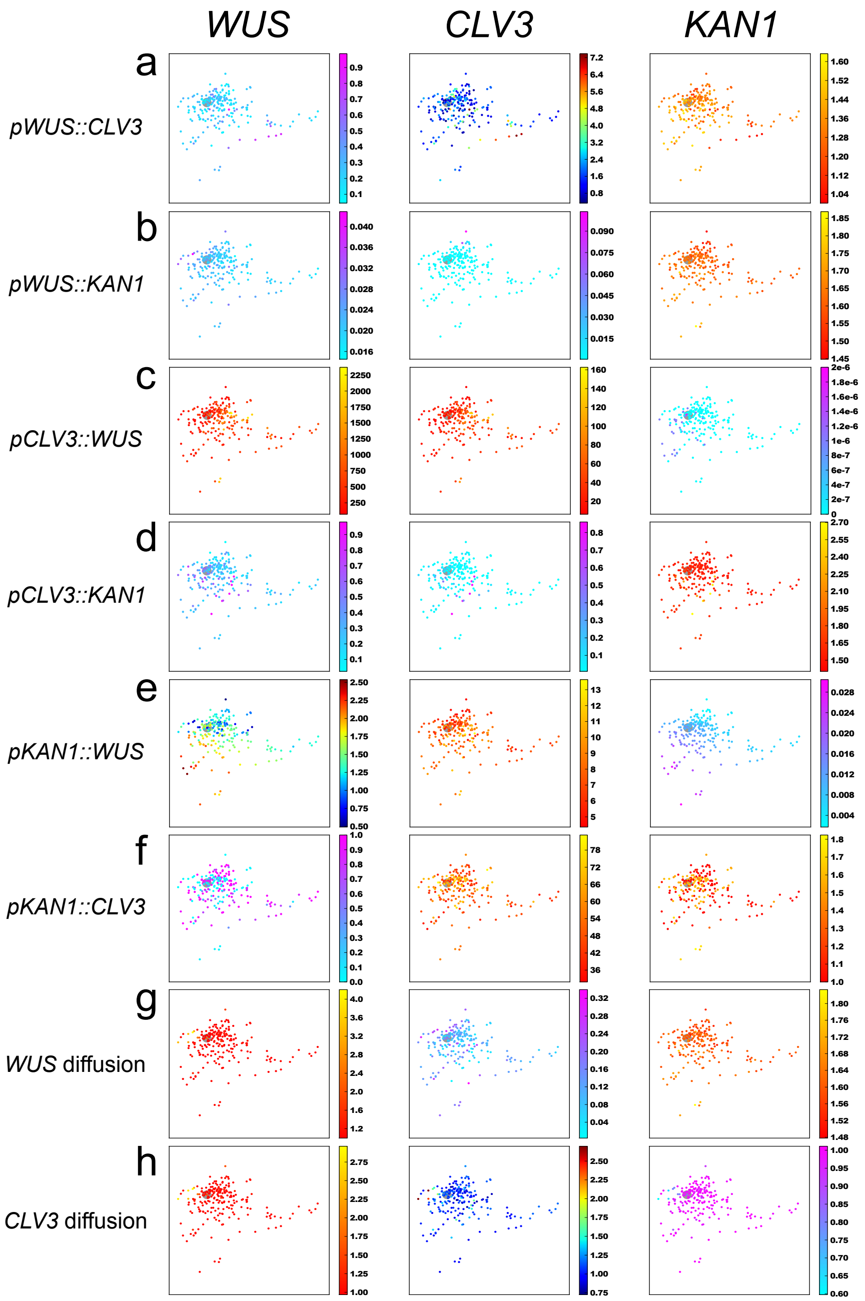

Supplementary Figure 12: **Mis-expression and diffusion mutants.** Parameter sets are displayed using principal component analysis (first and second principal directions, *cf.* Figure 5b in main text). The color scale represents the variation of the expression of the considered gene between the wild type and the considered mutant. The three columns show the change in OZ/*WUS* (left), CZ/*CLV3* (middle), and PZ/*KAN1* (right) expression. The grey disc is centered on the parameter set exemplified in Supplementary Figure 3 (Supplementary Table 8). a) mis-expression mutants for *pWUS::CLV3*, b) *pWUS::KAN1*, c) *pCLV3::WUS*, d) *pCLV3::KAN1*, e) *pKAN1::WUS*, and f) *pKAN1::CLV3*. g) Transport malfunction mutants for WUS and H) CLV3. See Supplementary Information for description of mutant implementations.

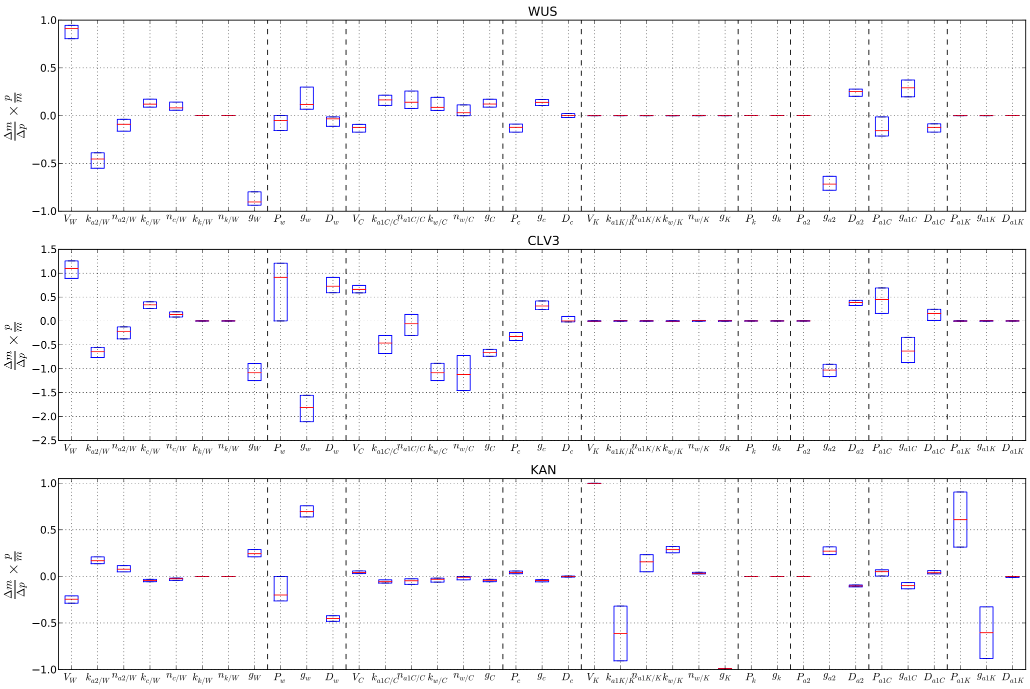

Supplementary Figure 13: **Sensitivity analysis.** For each gene, the sensitivity of the 229 sets to their parameter variations are plotted with boxplots, the median variation is marked with a red line, the extent of the boxes display the lower and upper quartiles of the data. The dashed lines separate the parameters directly affecting the involved species: from left to right, *WUS*, wus, *CLV3*, clv3, *KAN*, kan, a2, a1c and a1k, respectively.

3 Supplementary Tables (1-8)

Supplementary Table 1: **DEG Analysis of *WUS-GR* responses mapped to three cell types** In this table columns E and F represent DEX / MOCK set, columns G and H represent DEXCYC / CYC set for *35S::WUS-GR ap1-1;cal1-1* DEG analysis. Columns I-P display 3 SAM cell samples (CLV3p, FILp and WUSp) including the comparison of protoplasted ap1-1;cal1-1 SAMs (IND) with non-protoplasted SAMs (UNIND). All samples shows log2 fold change values and the adjusted p-value (FDR) of the LIMMA package (*e.g.* DEX_Mock_4hrs_fold_change and Dex_Mock_4hrs_adj_p-value). The GCRMA normalized expression values from Supplementary Table 5 were used for the DEG analysis. The column D provide 2 fold up regulated DEGs relative to 2 other samples for cell types. An overlap of *WUS-GR* data set with cell type data set is shown in Figure 2A-D. In addition, the table contains in column Q the detected genes for CLV3p, FILp and WUSp cell samples, respectively. For this MAS5 algorithm was used to make PMA calls, a genes scored as P in all the replicate was considered present and counted once (Supplementary Table 6).

Supplementary Table 2: ***WUS-GR* up and down regulated transcripts in the presence of Dex.** Down regulated genes were sorted from Supplementary Table 1 after applying ($\leq$ 2 fold; p $>$ 0.01).

Supplementary Table 3: ***WUS-GR* up and down regulated transcripts in Dex + cycloheximide.** Up regulated genes were sorted from Supplementary Table 1 after applying ($\geq$ 2 fold; p $>$ 0.01).

Supplementary Table 4: **GO Term Enrichment for *WUS* regulated transcriptome.** The table provides the GO term enrichment data for *WUS* regulated transcripts from Supplementary Table 3. The columns provide *WUS* up and down regulated set identifiers (DEG Sets), their sample sizes (Sample SZ), the GO identifier, the number of genes in the genome associated with a GO term (Node Size), the number of genes in the test sample associated with a GO term (Sample Match), the p-value of the hypergeometric distribution test (P-value), the Bonferroni corrected version of this p-value (P-value adjusted), the GO term, the Ontology type, and the Arabidopsis genes associated with a given GO term in the test sample (Sample AGIs).

Supplementary Table 5: **GCRMA Normalized Expression Values** The table provides GCRMA normalized expression values of CYC (cycloheximide), DEX (dexamethasone), DEXCYC (dexamethasone plus cycloheximide) and MOCK treated *35S::WUS-GR ap1-1;cal1-1* in columns (D-O). Three cell types (CLV3p, WUSp and FILp) GCRMA normalized expression values presented in columns (P-V). In addition, GCRMA normalized values for protoplasting induced and un induced replicates also provided in columns (X-AA).

Supplementary Table 6: **MAS5 Normalized Expression Values with Present Call Information** This table provides mean of MAS5 normalized expression values for three cell types with PMA calls. The present call information (PMA values) from the Wilcoxon signed rank test of the MAS5 algorithm is provided for the cell types in column (e.g. CLV3p_PMA calls). The probe set showing present calls in all the replicate of a sample was considered as positive.

Supplementary Table 7: **List of primers used in this study**

V_C	$0.6755^{+7.7405}_{-0.0484}$	$k_{a1c/C}$	$0.04344^{+0.4220}_{-0.0022}$	$k_{w/C}$	$0.08876^{+0.2225}_{-0.0045}$	g_C	0.1
P_c	0.1	g_c	0.1	D_c	$3.5429^{+4.8937}_{-0.0005}$	V_W	$0.2368^{+0.4025}_{-0.0536}$
$k_{a2/W}$	$0.0239^{+0.1255}_{-0.0014}$	$k_{c/W}$	$0.0473^{+6.6450}_{-0.0265}$	$k_{k/W}$	0.01	g_W	0.1
P_w	0.1	g_w	0.1	D_w	$0.0424^{+0.1134}_{-0.0132}$	V_K	$0.1887^{+0.3745}_{-0.0806}$
$k_{a1k/K}$	$0.1898^{+0.9541}_{-0.0082}$	$k_{w/K}$	$0.0039^{+0.0149}_{-0.0003}$	g_K	0.1	P_k	0.1
g_k	0.1	D_k	0	P_{a1c}	$0.0098^{+0.1639}_{-0.0007}$	g_{a1c}	0.1
D_{a1c}	$0.0245^{+0.0774}_{-0.0011}$	P_{a1k}	$0.0209^{+0.2600}_{-0.0027}$	g_{a1k}	0.1	D_{a1k}	$0.0008^{+0.0154}_{-1.1230e-5}$
P_{a2}	$0.0656^{+0.3325}_{-0.0035}$	g_{a2}	0.1	D_{a2}	$0.0071^{+0.0835}_{-0.0005}$	n	2

Supplementary Table 8: **Parameter values.** In red are the parameters where values have been obtained via optimization. The values are the ones used in all example simulations. The underscripted values precedeed by a minus sign are the minimal values in the set of 229 parameter values obtained in the optimization, the upperscripted values precedeed by a plus sign are the maximal values in this set.

4 Supplementary Movies (1-2)

Supplementary Movie 1: ***p35S::CLV3* simulation.** From left to right: *WUS* expression domain, *CLV3* expression domain, *KAN1* expression domain.

Supplementary Movie 2: **Growth and cell division simulation.** From left to right: *WUS* expression domain, *CLV3* expression domain, *KAN1* expression domain.
