# Supplementary material for: Plant stem cell maintenance involves direct transcriptional repression of differentiation program
Source: Mol Syst Biol. 2013 Apr 2;9:654. doi: 10.1038/msb.2013.8 (PMC3658276; doi:10.1038/msb.2013.8)
Supplement: Supplementary Table 7 — List of primers used in this study [file msb20138-s9.doc]

Supplementary Table 7

| **Name** | | | | | |
| --- | --- | --- | --- | --- | --- |
| **Gene Primer name Sequence** | | | | | |
| **qRT-PCR Primers** | | | | | |
| TUB | TUB2-F | | | | GAGCCTTACAACGCTACTCTGTCTG |
|  | TUB2-R | | | | GAAGACGGGGGAATGGGATGAGATT |
| KAN1 | SP/KAN1/1321-1570 | | | | GTAACTCTTCACGAGAGACATGGC |
|  | ASP/KAN1/1321-1570 | | | | ATAAAAATCCAAATTGATCAAGAA |
| KAN2 | SP/KAN2/1241-1461 | | | | AATTCTTCCGGAGAAGCACGTTTG |
|  | ASP/KAN2/1241-1461 | | | | ACTACCTAGCTACAACAATAAATT |
| AtGRF6 | SP/At2g06200/435-800 | | | | GCCTCCTCCTACTCAATTCACTCCAAATCT |
|  | ASP/At2g06200/435-800 | | | | ATGATCTCTTTGTTTCAGCTTCTCCTCTAA |
| AtMYB51 | SP/At1g18570/571-899 | | | | GAAGACGAAGAACGTTCAATCATCT |
|  | ASP/At1g18570/571-899 | | | | GATCATGGTCACTAGTTGTCAGATT |
| UFO | SP/At1g30950/411-700 | | | | CCGATGGTACCGTCTCTCTTTTGCT |
|  | ASP/At1g30950/411-700 | | | | CCAGCGACAGTAACATCAATAGAGG |
| AS2 | SP/At1g65620/401-700 | | | | GCAACCCCAAATAGCAGCTTAAAGA |
|  | ASP/At1g65620/401-700 | | | | ACACATTCCGGTTGACATTTTCGCC |
| AtHB22 | SP/At4g24660/601-895 | | | | GAACAGAAAGAGAAGATGTTAGCCT |
|  | ASP/At4g24660/601-895 | | | | GCAGAAGTAGTTACAGAAGAGTCAT |
| YAB3 | SP/At4g00180/901-1200 | | | | ACTGGGCTCATTTCCCTCACATACAC |
|  | ASP/At4g00180/901-1200 | | | | CCAAGAGAAAATTCTAGTAGTTCCGAAA |
| bHLH | SP/At1g511140/1211-1500 | | | | CAAGGACCTGCAAGAACAAGTGAA |
|  | ASP/At1g511140/1211-1500 | | | | CATTGGAGTTCGGACCATTACACA |
| KNAT3 | SP/At5g25220/1227-1490 | | | | TCTCATTCCAAATGGCCTTACCC |
|  | ASP/At5g25220/1227-1490 | | | | AAACCCTGCTTTCAAATCCCATC |
| unknown | SP/At5g65480/641-900 | | | | GAATGGCTTCTTCTCTCCTGCTG |
|  | ASP/At5g65480/641-900 | | | | TGGTGTTTTGTTGTCACTCGCTT |
| RLK | SP/At5g56040/2911-3200 | | | | CGATGGAGATTCGTCTAAATTGA |
|  | ASP/At5g56040/2911-3200 | | | | GCATCTCATGCATTATAGGATCT |
| unknown | SP/At3g47295/151-455 | | | | CACTTGTAACATCTTCATCCCCA |
|  | ASP/At3g47295/151-455 | | | | GGTTTTGCTTTGATACTCAGATG |
| MEE59 | SP/At4g37300/444-700 | | | | GAGTATTACTCAAATCTCACCGC |
|  | ASP/At4g37300/444-700 | | | | TTCGAAAGATCAGTTCAAGAACT |
| PAP2 | SP/At4g29080/953-1200 | | | | AAGAAGCTGAGAATCATGAAGAG |
|  | ASP/At4g29080/953-1200 | | | | AGTAAGAGGAAGAGGCCTTATTA |
| SCRM/ICE4 | SP/At5g65640/983-1269 | | | | GGCTTGGAGATTGAACAATGTGT |
|  | ASP/At5g65640/983-1269 | | | | CTCATAATGTTTTCCCCTAGGAA |
| DUF620 | SP/At5g06610/811-1096 | | | | GTGGCCTCTTAATTTGCCTTGAA |
|  | ASP/At5g06610/811-1096 | | | | GGAGGAATAAAACAATCAACGGA |
| RLK | SP/At4g34220/1791-2070 | | | | AATGGCAGCCTCCTCTGTTTCTT |
|  | ASP/At4g34220/1791-2070 | | | | TCGAAGTTGGTCCAGTGGTGTGA |
| KNAT1 | SP/At4g08150/1311-1600 | | | | TGGTGGGAGTTGCATTACAAGTG |
|  | ASP/At4g08150/1311-1600 | | | | GAAAGCAACGAGAGGTTGTTATT |
| bHLH | SP/At3g06590/697-925 | | | | ATGCAAGTGAGAGCCATGAACTC |
|  | ASP/At3g06590/697-925 | | | | GGGAAGACTCGGTTCAAAATTTA |
| SUC1 | SP/At1g71880/1442-1697 | | | | GCATCCATATTCTCAAGCTGCTC |
|  | ASP/At1g71880/1442-1697 | | | | ACTAGTGGAATCCTCCCATGGTC |
| AtHB13 | SP/At1g69780/721-1020 | | | | CTCATAATCAGAAACTCCAAGCT |
|  | ASP/At1g69780/721-1020 | | | | CCATACTCTGTCCTGAAGATGAG |
| AtbZIP44 | SP/At1g75390/478-769 | | | | GGGTCCTGCGTCTTAATCATAGT |
|  | ASP/At1g75390/478-769 | | | | GATCTGAGCGTTTTCTTTACGTA |
| **Primer for RNA probe synthesis used in *in situ* experiments** | | | | | |
| KAN1 probe | SP/KAN1/663-1969 | | | CACCGTCCAATTAGAGGCATCCCG | |
|  | ASP/KAN1/663-1969 | | | AAACCCTCTAGCCAACCAAGAGGC | |
| KAN2 probe | SP/KAN2/318-1461 | | | CACCAAACAGTAAACCATCATCGA | |
|  | ASP/KAN2/1241-1461 | | | ACTACCTAGCTACAACAATAAATT | |
| **ChIP-qRT-PCR Primers** | | | | | |
| KAN1-2000 | | Forward | | ACTGCAAGCATACTGTACTTC | |
| KAN1-2000 | | Reverse | | CTCGACAAATCATTAATGCCTTG | |
| KAN1-1000 | | Forward | | GAACAATCTACGGTCAGAAACTTG | |
| KAN1-1000 | | Reverse | | GACTGTGTCCTCTCTGACTCC | |
| KAN1 100 | | Forward | | ATCAAATCTTTCAGACACCC | |
| KAN1 100 | | Reverse | | TCTCTTGCTATTGCTGCCAC | |
| KAN1 800 | | Forward | | CAGTATGGAGTCGTTGGATCTTC | |
| KAN1 800 | | Reverse | | AGCTCAACAGCGTGAACAAAC | |
| KAN1 1500 | | Forward | | GGAAGAGATGGCACAATTGCC | |
| KAN1 1500 | | Reverse | | CTATCTATCATCTCAATTCTCC | |
| KAN2-1600 | | Forward | | GGCATTTGATTGGGTTTGGAC | |
| KAN2-1600 | | Reverse | | GAAGTTTACATTCGAAACTTC | |
| KAN2-400 | | Forward | | GCAATCCACTAATTATGAGACC | |
| KAN2-400 | | Reverse | | CTTTGGAGGAGAAAAGTAAATCC | |
| KAN2 700 | | Forward | | GACCAATAAGAGGAATACCAC | |
| KAN2 700 | | Reverse | | CTCTAACCGGAAACAACAATAG | |
| KAN2 1600 | | Forward | | CGTTTCTTCGTGTTAGATCTG | |
| KAN2 1600 | | Reverse | | CCTTTCTATTCTATGTGTGTCAG | |
| KAN2 2300 | | Forward | | CTTAACCATGGTTCCACTTCTCC | |
| KAN2 2300 | | Reverse | | AAAACTGTTCGGACACAGAAGC | |
| YAB3-650 | | Forward | | CATTAGTGCGACGACACAAAGTC | |
| YAB3-650 | | Reverse | | CAATGATGATAATGCAACAGTGG | |
| YAB3-100 | | Forward | | GAAACAAAGACTAGTTCCGGTTTG | |
| YAB3-100 | | Reverse | | TTGTAGAGATAAGCTGCATACC | |
| YAB3 900 | | Forward | | GGATAGATCTAAGTAGTTTGGC | |
| YAB3 900 | | Reverse | | GATGGCTCATCATCATGTTCATG | |
| YAB3 1500 | | Forward | | GAAGTGATGATGATAACGAGC | |
| YAB3 1500 | | Reverse | | TTGGTTTTTATTGTGCCTCCCC | |
| AS2-900 | | Forward | | CCCAGTAACATTAATCTCATAGG | |
| AS2-900 | | Reverse | | TACGGGAAAATTAGGAGATATACC | |
| AS2-400 | | Forward | | GTATGCAACCCCAAATAGCAGC | |
| AS2-400 | | Reverse | | GAATAAGAAAGCTTTTACCTGCGAG | |
| AS2 600 | | Forward | | CATATAGCTACACATATCTCTAGG | |
| AS2 600 | | Reverse | | CAGTTGACAAGAAGGTTGGATCAG | |
| AS2 1200 | | Forward | | GTTTCCTTATACCTTCTACCTC | |
| AS2 1200 | | Reverse | | GGTGAGTTTGTTGAAGAAGATG | |
| AS2 2100 | | Forward | | ATCACATACTTGCATTATAGAG | |
| AS2 2100 | | Reverse | | GGTCTATATTCTTGCTGGAGGTAC | |
| **Oligos Used For EMSA** | | | | | |
| KAN1-1100 | | Forward | | TACTGATATTTAATGTATATTTATCTCT | |
| KAN1-1100 | | Reverse | | AGAGATAAATATACATTAAATATCAGTA | |
| M1 | | Forward | | TACTGATATTTGGTGTATATTTATCTCT | |
| M1 | | Reverse | | AGAGATAAATATACACCAAATATCAGTA | |
| KAN1 1050 | | Forward | | TCTTACACTAATACTATATTTACACATG | |
| KAN1 1050 | | Reverse | | CATGTGTAAATATAGTATTAGTGTAAGA | |
| M2 | | Forward | | TCTTACACTGGTACTATATTTACACATG | |
| M2 | | Reverse | | CATGTGTAAATATAGTACCAGTGTAAGA | |
| KAN2 1080 | | Forward | | GGATTAATCGTTCACTTATTTTGTTT | |
| KAN2 1080 | | Reverse | | AAACAAAATAAGTGAACGATTAATCC | |
| M3 | | Forward | | GGATGGGTCGTTCACTTATTTTGTTT | |
| M3 | | Reverse | | AAACAAAATAAGTGAACGACCCATCC | |
| KAN2 1650 | | Forward | | TGTCAGTAATAGTAATATTGAGTA | |
| KAN2 1650 | | Reverse | | TACTCAATATTACTATTACTGACA | |
| M4 | | Forward | | TGTCAGTGGTAGTAATATTGAGTA | |
| M4 | | Reverse | | TACTCAATATTACTACCACTGACA | |
| M5 | | Forward | | TGTCAGTGGTAGTGGGGTTGAGTA | |
| M5 | | Reverse | | TACTCAACCCCACTACCACTGACA | |
| YAB3 500 | | Forward | | AGTTAATTAATGTGTGATATTAATTGGC | |
| YAB3 500 | | Reverse | | GCCAATTAATATCACACATTAATTAACT | |
| M10 | | Forward | | AGTTAATTGGTGTGTGATATTAATTGGC | |
| M10 | | Reverse | | GCCAATTAATATCACACACCAATTAACT | |
| M11 | | Forward | | AGTTGGTTGGTGTGTGATATTAATTGGC | |
| M11 | | Reverse | | GCCAATTAATATCACACACCAACCAACT | |
| M12 | | Forward | | AGTTGGTTGGTGTGTGATAGGGGTTGGC | |
| M12 | | Reverse | | GCCAACCCCTATCACACACCAACCAACT | |
| AS2 750 | | Forward | | CCAATATCTTTAATACTACTAATTGCAA | |
| AS2 750 | | Reverse | | TTGCAATTAGTAGTATTAAAGATATTGG | |
| M8 | | Forward | | CCAATATCTTTAATACTACTGGTTGCAA | |
| M8 | | Reverse | | TTGCAACCAGTAGTATTAAAGATATTGG | |
| M9 | | Forward | | CCAATATCTTTGGTACTACTGGTTGCAA | |
| M9 | | Reverse | | TTGCAACCAGTAGTACCAAAGATATTGG | |
| AS2 -480 | | Forward | | AACGTTAATCATCATAATTTCTATAACC | |
| AS2 -480 | | Reverse | | GGTTATAGAAATTATGATGATTAACGTT | |
| M6 | | Forward | | AACGTTGGTCATCATAATTTCTATAACC | |
| M6 | | Reverse | | GGTTATAGAAATTATGATGACCAACGTT | |
| M7 | | Forward | | AACGTTGGTCATCATGGGTTCTATAACC | |
| M7 | | Reverse | | GGTTATAGAACCCATGATGACCAACGTT | |
| **Primers Used For Reporter Gene Assay** | | | | | |
| KAN11050HIND | | | Forward | AAGCTTATCATCATGGGATGATCAGATC | |
| KAN11050BAMH | | | Reverse | GGATCCATCCATGAGCTCAAGAACCGAC | |
| KAN11050mut | | | Forward | GTGACCATGATATAAATGTGTAC | |
| KAN11050mut | | | Reverse | ATTCTAATGATCATTCCCATTTTTC | |
| **Primers Used For RTPCR** | | | | | |
| UBQ | | | Forward | TTCACTTGGTCCTGCGTCTTCGTGGTGGTTTC | |
| UBQ | | | Reverse | CAAGTCGAACTTCTTCTTTATCATCGCTTCG | |
| WUS | | | Forward | GCATATGCCCATGCAGAGACCTGCTAATTCCG | |
| WUS | | | Reverse | TGGAATTCCTATGCCCATCCTCCACCTACGTTG | |
| CLV3cds | | | Forward | ATGGATTCGAAGAGTTTTCTGCTACTAC | |
| CLV3cds | | | Reverse | TCAAGGGAGCTGAAAGTTGTTTCTTGGC | |
| CLV3 3’utr | | | Forward | AGTTTCTATATTTCTCTCTGTATC | |
| CLV3 3’utr | | | Reverse | GAAATAATTTAAAGCAACAAGAGA | |

**Table S7**. List of primers used in this study.
